# Animal Magnetism

**Published:** 1837

**Authors:** 


### Art. VII.—Animal Magnetism.

*Report of Dr. Franklin and other Commissioners charged by the King of France with the examination of Animal Magnetism as practised at Paris. Translated from the French; with an Historical Outline of the "Science;" an abstract of the Report on Magnetic Experiments, made by a Committee of the Royal Academy of Medicine, in* 1831, *and Remarks on Col. Stone's Pamphlet.* Philad. 1837. 2d edition.

*Practical Instruction in Animal Magnetism*, by J. P. F. Deleuze. Translated from the French, with notes, by Thos. C. Hartshorn, Providence, 1837.

*A letter to Dr. Brigham, on Animal Magnetism, describing an interview with Miss Loraina Brackett, while in a state of Somnambulism.* By William L. Stone. New-York: 1837.

*Exposition, or a New Theory of Animal Magnetism; with a Key to the Mysteries: demonstrated by experiments, with the most celebrated somnambulists in America: also, Strictures on Col. Stone's letter to Dr. Brigham.* By C. F. Durant. New-York: 1837.

Mr. Durant, in the last and latest of the works we have just cited, writes as if he were in a passion with all the advocates of Animal Magnetism, and, especially, with Col. Stone; and modestly claims to be regarded as a public benefactor, for having produced a book, the "withering influence of whose pages" will forthwith kill off the whole imposture! It would be cruel to rob Mr. Durant of the eminent gratification which his fancied achievement affords him, but of this we have no apprehension, should we ask, whence the special good to the community, if you *have* killed off the delusion? For ourselves, we perceive no great amount either of good or evil, connected with the art of Animal Magnetism. It is undoubtedly a copious source of excitement to all who concern themselves with it, either as impostors or impostorees, if so they should be called, and why not let them enjoy it?

In commencing this article, we write on the fence, reserving to ourselves to descend or not; and, should we do so, to select the field of credulity or of incredulity, according to their relative luxuriance. Our object is to supply our distant and insulated readers, with some account of what they may not have had the opportunity of knowing.

And first of all, we would disabuse their minds and our own, of the delusion, that there is any connexion between the phenomena of Magnetism and those of *Animal* Magnetism, so dubbed. When Mesmer revived and began to practice the art of bringing certain weak-minded men and women, with shattered nervous systems, into a peculiar state of mind and body, *apparently* resembling that, which is known under the name of somnambulism, or sleep-walking, he put forth as a theory, that a magnetic influence was exerted; and, as magnetism was at that time but little understood, its occult character, was well adapted to the taste of the lovers of mysticism; and his hypothesis, undoubtedly, contributed to the popularity of his art. In more modern times, as it has been discovered that magnetism plays an important part in the operations of nature, or is a modification of galvanism, the philosophical mind is not backward in imagining, that we are on the eve of some great development. Thus the word magnetism continues to exercise over us a kind of talismanic power, to which the credulous readily yield themselves up, and from which the most incredulous, find it somewhat difficult to keep themselves extricated.

But let us return to the origin and history of the art, which are briefly set forth in the following extract from the Encyclopædia Americana:

"Animal Magnetism originated thus: Anthony Mesmer, (q. v.) in 1772, attempted cures with the mineral magnet, and excited some sensation in Vienna, but at length declared, that not the magnet, but a mysterious power in his own person, caused the effects ascribed to the magnet, and that this power was related not only to the magnetic power, but to the attraction dispersed throughout the universe. But a fraud which he attempted (the pretended restoration of sight to a girl) having been discovered, he proceeded, in 1778, to Paris. The attention which he attracted there, and the final report of a committee of the academy on magnetism, or, as it is also called *Mesmerism*, we shall speak of under *Mesmer*. The great supporters of animal magnetism have recently been Kieser, in Jena, and Wolfart, in Berlin; the former explains the phenomena by the striking difference between life by day and life by night, both in the case of animals and vegetables; the latter adopts the mystical jargon of Mesmer. (See *Archives of Animal Magnetism*, by Kieser, Nasse, and Nees von Esenbeck, published since 1817, in numbers, and since 1825, under the title *Spinx, or New Archives of Animal Magnetism;* and Wolfart's *Annals of Animal Magnetism (Lebens-Magnetismus)*, 10 numbers, 1818, et seq.) In 1820, the Prussian government caused a prize to be offered for the best treatise on this subject, but it was subsequently withdrawn. Among the numerous works which treat of it, are Deleuze's *Histoire critique Magnetisme Animal*, (Paris, 1813); Jos. Ennemoser's *Der Magnetismus in einer Geschichtlichen Entwickelung von allen Zeiten und bei allen Volkern* (Leipsic, 1819), in the spirit of Mesmer and Wolfart; J. C. L. Ziermann's *Geschichtliche Darstellung des Thierischen Magnetismus als Heilmittel's* (Berlin, 1824), less prejudiced; *Del Magnetismo Animale*, by Basevi (Florence, 1826)."

In 1778, Mesmer left Germany, and repaired to Paris. He soon attracted public attention, and was visited by so many invalids, that he was under the necessity of employing assistants, one of whom was M. Deslon, whose name will reappear in the course of this review. The migration of Mesmer to Paris, led to inquiry into his art by M. Thouret, Regent Physician to the Faculty of Paris, and a member of the Royal Society of Medicine. The book published by this learned physician, entitled "*Enquiries and Doubts respecting the Animal Magnetism*," was submitted to the scrutiny of a committee of six members of the society, from whose report on its merits we make the following extract:

"This picture of the Animal Magnetism, as it was invented and applauded by the ancients, is faithfully extracted from the performance of M.

Thouret. The principal authors, to which he has recourse in the progress of his enquiry, are Paracelsus, Van Helmont, Goclenius, Burgravius, Libavius, Wirdig, Maxwell, Santanelli, Tentzel, Kircher, and Borel. The entire passages are extracted, and M. Thouret has displayed in this performance, as he had already done in so many others, an erudition the most various, the most precise, and the most extensive.

It is easy to see how analogous is the system we have described to that of M. Mesmer. To demonstrate this analogy, M. Thouret has considered separately each of the propositions published and avowed by the latter. They amount to twenty-seven, and the result of this examination is, that they are all positively announced in some of the authors whose names have been recited.

Every part of Mesmer's system, even down to the experiments of the ring and the sword, have been found by M. Thouret in the works of these writers. It is therefore certain, that the assertions of M. Mesmer, which are represented by him as principles of his own, do not belong to him, and that this theory, in the room of being an attractive novelty, is an ancient system, abandoned by the learned near a century ago."

In the same year, 1784, in which this Report was made, the practice of Animal Magnetism had excited so much interest in France, that the king directed an inquiry to be made, and appointed a board of nine Commissioners, four from the R. A. of Medicine and five from the R. A. of Science, to investigate and report on the alleged effects produced by the magnetisers.—Among these Commissioners were Lavoisier and our own Franklin, then Resident Minister in Paris. The Commissioners entered on their duty with the deliberation and sagacity of true philosophers, and the manner in which they conducted the inquiry, not less than that in which they drew up the account of its course and results, renders their report worthy of being read as a model of philosophical research and composition.

The Report sets out with the following extract from a Memoir of Mesmer, published in 1779.

"It [the Animal Magnetism] is a fluid universally diffused; the vehicle of a mutual influence between the celestial bodies, the earth, and the bodies of animated beings; it is so continued as to admit of no vacuum; its subtlety does not admit of illustration; it is capable of receiving, propagating, and communicating all the impressions that are incident to motion; it is susceptible of flux and reflux. The animal body is subject to the effects of this agent; and these effects are immediately produced by the agent insinuating itself into the substance of the nerves. We particularly discover in the human body qualities analogous to those of the load-stone; we distinguish it in poles different and opposite. The action and the virtue of the Animal Magnetism are capable of being communicated from one body to another, animated or inanimate; they exert themselves to considerable distances, and without the least assistance from any intermediate bodies: this action is increased and reflected by mirrors; it is communicated, propagated and augmented by sound; and the virtue itself is capable of being accumulated, concentrated and transferred. Though the fluid be universal, all animal bodies are not equally susceptible of it, there even are some, though very few, of so opposite a nature, as by their mere presence to supersede its effects upon any other contiguous bodies.

The Animal Magnetism is capable of curing immediately diseases of the nerves, and mediately other distempers; it improves the action of medicines: it forwards and directs the salutary crises so as to subject them totally to the government of the judgment; by means of it the physician becomes acquainted with the state of health of each individual, and decides with certainty upon the causes, the nature and the progress of the most complicated distempers; it prevents their increase, and effects their extirpation, without at any time exposing the patient, whatever be his age, sex, or constitution, to alarming incidents, or unpleasing consequences." "In the influence of the magnetism, nature holds out to us a sovereign instrument for securing the health and lengthening the existence of mankind."

It was the duty of the Commissioners to examine into the properties and effects of this agent. From some cause which does not appear, their intercourse, for this purpose, was not with Mesmer, himself, but with M. Deslon, already mentioned, his former pupil and partner. Deslon was a physician of Paris, and readily offered to the Commissioners every desirable facility for the prosecution of their object. The following is the account which they give of the processes employed and the effects produced:

"After having thus made themselves acquainted with the theory and practice of the Animal Magnetism, it was necessary to observe its effects. For this purpose the commissioners adjourned themselves, and each of them repeatedly witnessed the public method of M. Deslon. They saw in the centre of a large apartment a circular box, made of oak, and about a foot or a foot and a half deep, which is called the bucket; the lid of this box is pierced with a number of holes, in which are inserted branches of iron, elbowed and moveable. The patients are arranged in ranks about this bucket, and each has his branch of iron, which by means of the elbow may be applied immediately to the part affected; a cord passed round their bodies connects them one with the other: sometimes a second means of communication is introduced, by the insertion of the thumb of each patient between the forefinger and thumb of the patient next him; the thumb thus inserted is pressed by the person holding it; the impression received by the left hand of the patient communicates through his right, and thus passes through the whole circle.

A piano forte is placed in one corner of the apartment, and different airs are played with various degrees of rapidity; vocal music is sometimes added to the instrumental.

The persons who superintend the process have each of them an iron rod in his hand, from ten to twelve inches in length.

M. Deslon made to the commissioners the following declarations:—1st. That this rod is a conductor of the magnetism, has the power of concentring it at its point, and of rendering its emanations more considerable. 2dly. That sound, conformably to the theory of M. Mesmer, is also a conductor of the magnetism, and that to communicate the fluid to the piano forte, nothing more is necessary than to approach to it the iron rod; that the person who plays upon the instrument furnishes also a portion of the fluid, and that the magnetism is transmitted by the sounds of the surrounding patients. 3dly. That the cord which is passed round the bodies of the patients is destined, as well as the union of their fingers, to augment the effects by communication. 4thly. That the interior part of the bucket is so constructed as to concentrate the magnetism, and is a grand reservoir, from which the fluid is diffused through the branches of iron that are inserted in its lid.

The commissioners, in the progress of their examination, discovered, by means of an electrometer and a needle of iron not touched with the loadstone, that the bucket contained no substance either electric or magnetical; and from the detail that M. Deslon has made to them respecting the interior construction of the bucket, they cannot infer any physical agent, capable of contributing to the imputed effects of the magnetism.

The patients then, arranged in considerable number and in successive ranks round the bucket, derive the magnetic virtue at once from all these conveyances: from the branches of iron, which transmit to them that of the bucket; from the cord which is passed round their bodies, and the union of their fingers, which communicate to them that of their neighbors; and from the sound of the piano forte, or of a musical voice, which diffuses it through the air. The patients are beside magnetised directly, by means of a finger or a bar of iron, guided before the face, above or behind the head, and over the surface of the part affected, the distinction of the poles still observed; they are also acted upon by a look, and by having their attention excited. But especially they are magnetised by the application of the hands, and by the pressure of the fingers upon the hypochonders and the regions of the lower belly; an application frequently continued for a long time, sometimes for several hours.

In this situation the patients offer a spectacle extremely varied, in proportion to the different habits of body. Some of them are calm, tranquil, and unconscious of any sensation; others cough, spit, are affected with a slight degree of pain, a partial or an universal burning, and perspirations; a third class are agitated and tormented with convulsions. These convulsions are rendered extraordinary by their frequency, their violence and their duration. As soon as one person is convulsed, others presently are affected by that symptom. The commissioners saw accesses of this kind, which lasted upwards of three hours; they were accompanied with expectorations of a thick and viscous water, brought away by the violence of the efforts. Sometimes these expectorations were accompanied with small quantities of blood; and there is among others a lad, a patient, who has frequently brought up blood in considerable abundance. These convulsions are characterised by precipitate and involuntary motions of all the limbs or of the whole body, by a contraction of the throat, by sudden affections of the hypochonders and the epigastrium, by a distraction and wildness in the eyes, by shrieks, tears, hiccupings, and immoderate laughter. They are either preceded or followed by a state of langour and reverie, by a species of dejection, and even drowsiness. The least unforeseen noise occasions starting; and it has been observed, that the changing of the key and the time, in the airs played upon the piano forte, had an effect upon the patients; so that a quicker motion agitates them more, and renews the vivacity of their convulsions.

Those who have acquainted themselves with the means and effects of the magnetism of the present day, of which we shall come in due time to say something, will perceive how they differ from those of Mesmer and his immediate followers. To the true character of these effects the Commissioners immediately directed their inquiries. They admitted that under the circumstances in which Deslon placed his patients, some of them might get well, a part from the restorative powers of nature acting at the same time, and others from the force of imagination; but they would not allow that cures should be received as evidence of the existence of a special agent, and determined, therefore, to institute a series of experiments, the first of which was on themselves; and we give the results in the following extract:

"In pursuance of these determinations, a particular apartment and a separate bucket were destined for their use in the house of M. Deslon, and the commissioners repaired thither once in the course of every week. The operation was continued in each experiment for two hours and a half, the branch of iron being in contact with the left hypochonder, surrounded with a cord of communication, and forming from time to time the chain of fingers and thumbs. They were magnetised either by M. Deslon, or in his absence, by one of his pupils, some of them for a longer time, and more frequently than others, and those with whom this was the case were the commissioners who appeared from constitution and habit the most susceptible. The operation was performed sometimes with the finger and the rod of iron, presented and guided along the different parts of the body, sometimes by the application of the hands and the pressure of the fingers, either upon the hypochonders or upon the pit of the stomach.

Not one of the commissioners felt any sensation, or at least none which ought to be ascribed to the action of the magnetism.

The commissioners, having at first submitted to the experiment only once a week, were desirous to ascertain whether a continuity of experiment would produce any effect: they submitted to it three days successively, but their insensibility was the same, and the magnetism appeared with respect to them perfectly impotent. This experiment, made at once upon eight different subjects, several of whom were subject to habitual derangements of health, authorises the conclusion that the magnetism has little or no action in a state of health, or even in a state of lesser infirmity."

The Commissioners next proceeded to try experiments on others, and for this purpose assembled at Passy, in the house of Dr. Franklin, who was at that time confined by ill health.

Seven patients were chosen from among the lower classes, and the operation was performed upon them by Mons. Deslon. Of these seven, four felt no sensation whatever,—three only experienced any effects, and these were inconsiderable. Their occurrences, however, determined the Commissioners to continue the experiments.

They now chose patients from the more intelligent classes, who were better qualified to narrate their feelings. Two ladies and two gentlemen were placed at the *bucket*, where they were kept for an *hour and nineteen minutes*. One of each sex felt nothing; the other gentleman thought he experienced a slight sensation in his swelled knee, when the hand was passed over it; and the other lady (subject to nervous disorders) had a feeling of irritation and dejection, with a disposition to sleep.

"Dr. Franklin, though the weakness of his health hindered him from coming to Paris, and assisting at the experiments which were there made, was magnetised by Mons. Deslon at his own house at Passy. The assembly was numerous; every person who was present underwent the operation. Some sick persons, who had come with M. Deslon, were subject to the effects of the magnetism in the same manner as at the public process; but Madame de B——, Dr. Franklin, his two relations, his secretary, and an American officer, felt no sensation, though one of Dr. Franklin's relations was convalescent, and the American officer had at that time a regular fever."

The Commissioners were now attracted to Dr. Jumelin, another magnetiser, whose method was nearly the same with that of Mesmer.

"Eight men and two women submitted to the operation in the first experiment, and felt nothing; at length a woman, who waits in the hall of M. Alphonse le Roy, doctor of physic, having been magnetised in the forehead, but without touching her, said that she felt the sensation of heat. M. Jumelin guiding his hand, and presenting the five extremities of his fingers over the whole of her face, she said that she felt as it were a flame, that passed from place to place; magnetised in the stomach, she said that she felt heat; magnetised upon the back, she made the same declaration; she also said that she felt hot in every part of her body, and that her head ached.

The commissieners, observing that, of eleven persons that underwent the experiment, one only had been sensible to the magnetism of M. Jumelin, were of opinion that this person had experienced certain sensations, only because she had probably an imagination more easily excited than the rest: the opportunity was favorable for clearing up the point. The sensibility of this woman being perfectly established, the business was only to protect her from the illusions of the imagination, or at least to leave her imagination without any thing to direct its operations. The commissioners proposed to blindfold her, in order to observe what her sensations would be, when she could no longer know any thing respecting the conduct of the experiment. She was accordingly blindfolded and magnetised; the phenomena no longer answered to the places towards which the magnetism was directed. Magnetised successively upon the stomach and in the back, she felt only a heat in her head, a pain in both eyes and in the left ear.

The bandage was removed from her eyes, and M. Jumelin having applied his hands upon the hypochonders, she said that she felt heat; after a few minutes she said that she was ready to faint, and she fainted in effect. When she was tolerably recovered, the experiment was resumed; she was blindfolded, M. Jumelin was removed, silence recommended, and the woman was induced to believe that the operation was performing. The effects were the same, though no operation, either near or distant, was performed; she felt the same heat, the same pain in her eyes and in her ears; besides which she felt a heat in her back and loins.

After a quarter of an hour, a sign was made to M. Jumelin to magnetise her in the stomach, she felt no sensation; in the back, it was the same thing. The sensations diminished instead of augmenting. The pains in her head continued, the heat in her back and loins ceased."

Believing that nervous patients might experience sensations in the parts to which their attention and imagination were directed from their observing the movements of the hands of Dr. J., the Commissioners proposed to him to perform the operation when their eyes were blindfolded. Meeting at the house of the operator—

"They began with an experiment upon his servant. They fixed a bandage over his eyes, prepared for the purpose, and which they employed in all the succeeding experiments. The bandage was made of two calottes of elastic gum, whose concavity was filled with edredon; the whole enclosed and sewn up in two pieces of stuff of a circular form. These pieces of stuff were then fastened to each other, and to two strings which were tied in a knot at the back part of the head. Placed over the eyes, they left in their interval room for the nose, and the entire liberty of respiration, without the person blindfolded being permitted to receive even the smallest particle of light, either through, or above, or below the bandage. These precautions having been contrived, with an equal view to the convenience of the subject, and the certainty of the result, the servant of M. Jumelin was persuaded that the operation was performing upon him. Upon this he felt an almost universal sensation of heat, and certain emotions in the region of the belly, together with an extreme heaviness: by degrees he grew drowsy, and appeared upon the point of falling asleep. This experiment proves what we have already said, that the symptom of drowsiness is the effect of situation and weariness, not of the magnetism.

The same person being afterwards magnetised with his eyes uncovered, and a rod of iron being presented to his forehead, he experienced sensations of pricking: the bandage being then replaced and the circumstance repeated, he was conscious to no sensation. The rod of iron was then removed, and the patient being interrogated if he felt nothing in his forehead, he declared that he felt something move backward and forward from one side of it to the other."

Having performed many other experiments with the same result, the Commissioners became convinced, that the imagination was in reality the chief if not the only source of all the effects produced; and they resolved to reduce the supposition to the test of experiment. They again had recourse to Deslon, who informed them, that when a tree is magnatized, it will, on being approached, impart its influence. He himself chose his subject, a boy, 12 years of age. An apricot tree in the orchard of Dr. Franklin, was selected, sufficiently remote from other trees to prevent their drawing off its magnetism, and while this was imparted by Deslon, the boy was kept confined in the house. The operator was stationed at a certain distance from the tree, with his cane and countenance directed towards it, in order that the influence might be kept up.

"The boy was then brought into the orchard, his eyes covered with the bandage, presented successively to four trees upon which the operation had not been performed, and caused to embrace each of them for the space of two minutes, the mode of communication which had been prescribed by M. Deslon himself.

M. Deslon, present, and at a considerable distance, directed his cane towards the tree which had been the object of his operations.

At the first tree the boy being interrogated at the end of a minute, declared that he perspired in large drops; he coughed, spit, and complained of a slight pain in his head; the distance of the tree which had been magnetised was about 27 feet.

At the second tree he felt the sensations of stupefaction and pain in his head; the distance was 36 feet.

At the third tree the stupefaction and head-ache increased considerably; he said that he believed he was approaching to the tree which had been magnetized; the distance was then about 38 feet.

In fine, at the fourth tree, which had not been rendered the object of the eperation, and at the distance of about twenty-four feet from the tree which had, the boy fell into a crisis; he fainted away, his limbs stiffened, and he was carried to a neighboring grass-plot, where M. Deslon hastened to his assistance, and recovered him."

Many other experiments, followed by similar results, were performed, of which the following may be taken as a specimen. It was executed at the house of Dr. Franklin, to which Deslon had brought two female patients.

"Dame P—— had films over her eyes; but as she could always see a little, the bandage already described was employed. She was persuaded that M. Deslon had been brought into the room to perform the magnetical operation; silence was recommended; three commissioners were present, one to interrogate, another to make minutes of the transaction, and the third to personate M. Deslon. The conversation was pretended to be addressed to M. Deslon; he was desired to begin the operation; the three commissioners in the mean time remained perfectly quiet and solely occupied in observing her symptoms. At the end of three minutes, the patient began to feel a nervous shuddering; she had then successively a pain in the back of her head, in her arms, a creeping in her hands, (that was her expression) she grew stiff, struck her hands violently together, rose from her seat, stamped with her feet: the crisis had all the regular symptoms. Two other commissioners, who were in the adjoining room, with the door shut, heard the stamping of the feet and the clapping of the hands, and without seeing any thing, were witnesses to this noisy experiment.

The two commissioners we have mentioned were with the other patient, Mademoiselle B——, who was subject to nervous distempers. No bandage was employed upon her, but her eyes were at liberty; she was seated with her face towards a door which was shut, and persuaded that M. Deslon was on the other side, employed in performing upon her the magnetical operation. This had scarcely taken place a minute, before she began to feel the symptom of shuddering; in another minute she had a chattering of the teeth and an universal heat; in fine, in the third minute, she fell into a regular crisis. Her respiration was quick, she stretched out both her arms behind her back, twisting them extremely, and bending her body forward: her whole body trembled; the chattering of her teeth became so loud that it might be heard in the open air; she bit her hand, and that with so much force, that the marks of the teeth remained perfectly visible."

We shall not tire our readers with a further citation of experiments, all of which were attended with the same result; but present them with the conclusion to which the Commissioners were unanimously and irresistably brought.

"Compression, imagination, imitation, are therefore the true causes of the effects attributed to this new agent, known by the appellation o

Animal Magnetism, this fluid, which is said to circulate through the human body, and to be communicated from individual to individual. Such is the result of the experiments of the commissioners, and the observations they made upon the means employed and the effects produced. This agent, this fluid, has no existence."

In this conclusion the public so far acquiesced, that Animal Magnetism seemed killed off. Mesmer left Paris, and after residing for a time in England, in disguise, returned to Germany, and died in his native town, in the year 1815.

The Magnetism, however, was but in a state of suspended animation. A lucky discovery of the Marquis de Puysegur, reproduced the curiosity of the public mind. Happening to put a question to a patient whom he had thrown into a magnetic sleep, to his great surprise it was answered, and the *proper* method of treatment immediately pointed out, indicating that the patient had acquired a new faculty—that of practising physic on infallible principles! With this discovery, the number of magnetizers immediately increased, and the attention of all concerned was directed upon *Somnambulism*, as the prominent effect. The convulsive muscular crises of the Mesmerian Magnetism no longer appeared; and what is not less singular, the means employed were entirely different from those which were in use when Franklin and his French associates, carried on their investigations. To these means we shall first dirct the reader's attention.

In 1813, Joseph Philip Francis Deleuze, a native of Sisteron, in the Lower Alps, published at Paris a "Critical History of Animal Magnetism;" and in 1825 put forth a "Manual of Practical Instructions," the title of which is placed at the head of this article. From the latter work, which is regarded as of high authority by the faithful, and is, unquestionably, a most elaborate performance, we shall extract what is necessary to disclose the manipulation to such of our friends as may be disposed to practise it.

"Cause your patient to sit down in the easiest position possible, and place yourself before him, on a seat a little more elevated, so that his knees may be between yours, and your feet by the side of his. Demand of him in the first place that he give himself up entirely, that he think of nothing, that he do not trouble himself by examining the effects which he experiences, that he banish all fear, and indulge hope, and that he be not disquieted or discouraged if the action of magnetism produces in him temporary pains.

After you have brought youself to a state of self-collectedness, take his thumbs between your two fingers, so that the inside of your thumbs may touch the inside of his. Remain in this situation five minutes, or until you perceive there is an equal degree of heat between your thumbs and his: that being done, you will withdraw your hands, removing them to the right and left, and waving them so that the interior surface be turned outwards, and raise them to his head; then place them upon his two shoulders, leaving them there about a minute; you will then draw them along the arm to the extremity of the fingers, touching lightly. You will repeat this *pass** five or six times, always turning your hands and sweeping them off a little, before reascending: you will then place your hands upon the head, hold them there a moment, and bring them down before the face, at the distance of one or two inches, as far as the pit of the stomach: there you will let them remain about two minutes, passing the thumb along the pit of the stomach, and the other fingers down the sides. Then descend slowly along the body as far as the knees, or farther; and, if you can conveniently, as far as the ends of the feet. You may repeat the same processes during the greater part of the sitting. You may sometimes draw nearer to the patient so as to place your hands behind his shoulders, descending slowly along the spine, thence to the hips, and along the thighs as far as the knees, or to the feet. After the first passes you may dispense with putting your hands upon the head, and make the succeeding passes along the arms, beginning at the shoulder: or along the body, commencing at the stomach.

When you wish to put an end to the sitting, take care to draw towards the extremity of the hands, and towards the extremity of the feet, prolonging your passes beyond these extremities, and shaking your fingers each time. Finally, make several passes transversely before the face, and also before the breast, at the distance of three or four inches: these passes are made by presenting the two hands together and briskly drawing them from each other, as if to carry off the superabundance of fluid with which the patient may be charged. You see that it is essential to magnetize, always descending from the head to the extremities, and never mounting from the extremities to the head. It is on this account that we turn the hands obliquely when they are raised again from the feet to the head. The descending passes are magnetic, that is, they are accompanied with the intention of magnetizing. The ascending movements are not. Many magnetizers shake their fingers slightly after each pass. This method, which is never injurious, is in certain cases advantageous, and for this reason it is good to get in the habit of doing it.

Although you may have at the close of the sitting taken care to spread the fluid over all the surface of the body, it is proper, in finishing, to make several passes along the legs from the knees to the end of the feet. These passes free the head. To make them more conveniently, place yourself on your knees in front of the person whom you are magnetizing.

I think it proper to distinguish the passes that are made without

---

*I employ here the word pass, which is common to all magnetizers: it signifies all the movements made by the hand in *passing* over the body, whether by slightly touching, or at a distance.

touching, from those which are made with the touch, not only with the ends of the fingers, but with all the extent of the hand, employing at the same time a slight pressure. I give to these last the name of *magnetic frictions:* they are often made use of to act better upon the arms, the legs, and the back, along the vertebral column.

This manner of magnetizing by longitudinal passes, directing the fluid from the head to the extremities, without fixing upon any part in preference to others, is called *magnetizing by the long pass*, (magnétiser à grands courans.) It is more or less proper in all cases, and it is requisite to employ it in the first sitting, when there is no special reason for using any other. The fluid is thus distributed into all the organs, and it accumulates naturally in those which have need of it. Besides the passes made at a short distance, others are made, just before finishing, at the distance of two or three feet. They generally produce a calm, refreshing and pleasureable sensation."

This lengthened extract, however, contains but a small part of the minute instructions embraced in the hundred closely printed pages, which are to govern both the magnetiser and the magnetisee; and which are delivered with a circumstantiality, that cannot be travelled through, without a *feeling* of reality in the power and effects of the magnetism. Withal, M. Deleuze writes like a most benevolent man, and the conviction is unavoidable, that he is, himself, a firm believer. Every where he condemns a public exhibition of the phenomena, for the gratification of an idle curiosity; and insists that the new and mysterious power should be employed solely for the relief of the sick. Indeed he seems to intimate, that Magnetism exercises no influence over persons in health. But what is this power? Let M. Deleuze answer for himself.

"As we cannot comprehend how a body can act upon another at a distance, without there being something to establish a communication between them, we suppose that a substance emanates from him who magnetizes, and is conveyed to the person magnetized, in the direction given it by the will. This substance, which sustains life in us, we call the magnetic fluid. The nature of this fluid is unknown; even its existence has not been demonstrated; but every thing occurs as if it did exist, and that warrants us in admitting it, while we are indicating the means of employing magnetism.

If the will is necessary to direct the fluid, belief is necessary to induce one to make a firm and steady use of the faculties he possesses. Confidence in the power we possess, makes us act without effort and without distraction. As to the rest, confidence is only the consequence of belief: it differs in this only—one believes *himself* to be endowed with a power, whose reality he does not doubt.

Thus the first cendition of magnetizing is the will; the second is the confidence which the magnetizer has in his own powers; the third is benevolence, or the desire of doing good. One of these qualities may supply the others to a certain point; but to have the action at the same time energetic and salutary, the three conditions must be united.

Magnetism, or the action of magnetism, springs from three things: 1st. the will to act; 2d. a sign, the expression of that will; 3d. confidence in the means employed. If the desire of doing good be not united to the will to act, there will be some effects, but these effects will be irregular.

The faculty of magnetizing exists in all persons; but all do not possess it in the same degree. This difference of magnetic power in various individuals arises from the superiority which some have over others, in moral and physical qualities.

Therefore there are men who have a magnetic power very superior to that of others. It is so great in some persons, that they are obliged to moderate it.

The magnetic virtue develops itself by exercise, and a person uses it with more facility and success, when he has acquired the habit of exerting it."

Such is the theory, which it will be seen is not quite so physical as that of Mesmer. Now for the effects produced on the new and improved method.

"The magnetized person perceives a heat escaping from the ends of your fingers, when you pass them at a little distance before the face, although your hands appear cold to him, if you touch him. He feels this heat through his clothes, in some parts, or in all parts of his body before which your hands pass. He often compares it to water moderately warm, flowing over him, and this sensation precedes your hand. His legs become numb, especially if you do not carry your hands as low as his feet; and this numbness ceases when, towards the close, you make passes along the legs to the toes, or below them. Sometimes, instead of communicating heat, you communicate cold; sometimes, also, you produce heat upon one part of the body, and cold upon an other. There is often induced a general warmth, and a perspiration more or less considerable. Pain is felt in the parts where the disease is seated. These pains change place, and descend.

Magnetism causes the eyes to be closed. They are shut in such a manner that the patient cannot open them; he feels a calm, a sensation of tranquil enjoyment; he grows drowsy, he sleeps; he wakes when spoken to, or else he wakes of himself at the end of a certain time, and finds himself refreshed. Sometimes he enters into Somnambulism, in which state he hears the magnetizer, and answers him without awaking.

As the state of somnambulism ought entirely to change the manner of magnetizing, and as it does not take place except in a small number of cases, we will speak of it in a chapter by itself. Now, we are merely describing what occurs when there is no somnambulish, and pointing out the conduct to be observed in various circumstances."

Passing over a multitude of anomalous and topical effects observed by our author in a practice of 40 years, and detailed with astonishing gravity and minuteness, we shall proceed to his fourth chapter, which treats with equal patience of SOMNAMBULISM and CLAIRVOYANCE, or the faculty of seeing through opaque bodies; and that, too, not with the eyes only, but with various parts of the body, especially the epigastrium and occiput.

"Magnetic Somnambulism, which we call, simply, *Somnambulism*, because that term cannot be equivocal in this work, is a mode of existence during which the person who is in it, appears to be asleep. If his magnetizer speaks to him, he answers without waking; he can also execute various movements, and when he returns to the natural state, he retains no remembrance of what has passed. His eyes are closed; he generally understands those only who are put in communication with him. The external organs of sense are all, or nearly all, asleep; and yet he experiences sensations, but by another means. There is roused in him an internal sense, which is perhaps the centre of the others, or a sort of instinct, which enlightens him in respect to his own preservation. He is subject to the influence of his magnetizer, and this influence may be either useful or injurious, according to the disposition and the conduct of the magnetizer.*

Somnambulism presents phenomena infinitely varied. A description of them may be found in a great number of works published upon this subject. This is not the place to describe them. My design is solely to teach the means of obtaining the most useful results from this crisis, without exposing one's self to the least inconvenience.

Of all the discoveries which have excited attention, from the remotest antiquity, that of Somnambulism certainly gives us the most insight into the nature and the faculties of man. The phenomena to which it has drawn our attention, demonstrate the distinction of two things; the two-fold existence of the *internal* and the *external* man in a single individual: they offer a direct proof of the spirituality of the soul: they make evident the truth known to ancient sages, and so well expressed by M. de Bonald, that *man is an intelligence served by organs*. This advantage cannot be too highly appreciated, especially in an age when audacious minds do not fear to employ the researches of physiology to shake the certainty of the interior sentiment which reveals to us the dignity of man, his supremacy in the order of creation, and his moral liberty; a sentiment which is the basis of social life, and which engages to the practice of virtue, by pointing out to us in a future life the development of our earthly existence, and the recompense of sacrifices made to obey the dictates of conscience. On the other hand, Somnambulism makes known to us the means of curing diseases which are curable, and of relieving those which are not: it serves to rectify the errors of medicine as well as those of metaphysics; finally, it points out the origin of a great number of opinions prevalent anterior to the experiments which have confirmed their correctness: and it res-

*There are exceptions to the character here given, but they are extremely rare.

tores to the order of nature, a multitude of facts which philosophers have disdained to examine, either because ignorance and credulity had altered some of their circumstances, or because, in the dark ages, they were made to serve as the foundation of superstition."

Since we began the preparation of this Review, we have received the London Lancet for September the 9th, of the present year, and find in it a Clinical Lecture on Animal Magnetism, by the distinguished Dr. Elliotson, delivered in University College Hospital, from which we make the following extract. After speaking of some attempts of Mr. Chenevix, in 1829, to display to him (Dr. E.) the effects of Animal Magnetism, the reporter of the lecture continues:

"He was satisfied that there was something more than imagination in these things, but he had had no opportunity or time to carry on the investigation, till he heard of Baron Dupotet being in London, who had magnetised for twenty years, and some of whose works on magnetism he (Dr. E.) had read. He was determined to ask him to afford his assistance. The results of the experiments had been the following:—Generally speaking, it took no effect on male subjects, or if it did it was very slight, consisting of slight twitchings of the muscles, a feeling of fulness, and gasping, or catching of the breath,—in many cases not even so much effect as this. These effects were not, however, imaginary; he (Dr. E.) had felt them, and he had expected rather to go to sleep, if there had been any effect. Some, however, went to sleep, who had made up their minds that no effect could be produced, and had determined to resist it if possible. A great number of female patients had been sent to sleep, and so had one male epileptic for the space of ten minutes, not longer; he was, however, decidedly asleep. This effect was all that was produced in him, and this did not always occur. The experiments had been tried on three girls, one of them epileptic, and two of them hysterical. In the first case the girl was sent into a state of decided coma; she had no sensation; she was pricked with pins, and suffered no pain; she did not feel when her hair was pulled. During the time the influence lasted, she kept rolling her eyes, or moving her lower lip up and down; when the eyes were still the lip moved, and vice versa. Nothing but coma was produced in this case, but she could not open her eyes by herself, nor by transverse movements across them, except these movements were made by the magnetiser himself, as no one else in performing this manipulation succeeded. This was tried repeatedly, and found always to be the case, while the magnetiser succeeded instantly.

With regard to the patient he first mentioned, the effects were most decided; she did not fall into the ecstacy directly after she had been magnetised, but the change in the fits came on after she had been magnetized several times. Hence it was not an immediate effect of the process, though it had been produced by it. These paroxysms were, however, decidedly put an end to by magnetism. She would sit during the manipulations, and talk as ramblingly as possible, and be exceedingly abusive, then all at once she would stare about her, close her eyes for a moment, and be quite well again. Now, she was brought out of the attacks so repeatedly and so decidedly, that there could be no doubt that the magnetism produced the effects. There was no imagination acting here, as they had seen her when she sat down, restless and abusive, not knowing that she was to be brought to herself; indeed not knowing that she was not herself. She only came out of the fits once or twice without magnetism, but was brought out of them three-fourths of the times by that agency, when it was employed, and she had never, he believed, been brought out of the extatic delirium without it. Many persons had been convinced of the effects of magnetism by this case, who had not believed in it when only sleep was produced. There was no collusion, he felt convinced, in this case. Another patient, a girl, had been sent into a state of coma by means of magnetism; no pricking with pins, or pulling of her hair, would bring her out of that state. There was, therefore, no doubt that a coma, similar to that of epilepsy, could be produced by magnetism. Her jaw was so fixed that it could not be drawn down by the force of the hand, but when a few transverse motions were made over it, it gave way directly. This girl could not open her eyes until they had been magnetized. There was no deception practised in these things. Baron Dupotet said he would open one sense while the others remained shut. He placed his finger in one of her ears, and she heard slightly at first; she then began to hear better, and gradually got to the full sense of hearing, and answered questions; and the last time not only spoke, but fell into a violent rage, and shook an individual, who had offended her, with great force; she sat down, looking the picture of rage, her lips white, and she trembling all over with passion. She was awoke by a few transverse motions over the eyes, and knew nothing of what had occurred during the state of insensibility.

Now, so far as these facts had gone, that is, those that had come under his own notice, he, (Dr. E.) believed in what he should call Mesmerism, for Mesmer might be considered the second founder of the system. He (Dr. E.) was never ashamed to declare what he believed; he had little respect for authority, when he saw facts like those he had observed in the cases manipulated on by Baron Dupotet; he must believe them. The whole profession might laugh, but he must believe that there was a peculiar power which gave rise to the phenomena which he had observed, and that it was not sufficiently known or appreciated."

In illustration of what is here set forth, we shall extract from a brief history of Animal Magnetism, contained in one of the works, the title of which is placed at the head of this article, the following paragraph:

"The patients subjected to fits, predicted, in their state of somnambulism, the exact day and hour when they should have a return of the paroxysm, and as might very naturally be expected, the fits came on at the time. In this state they also were said to possess the same power of internal inspection with regard to other persons who were placed in magnetic connection *(en rapport)* with them. This condition was called the fifth degree, and all subsequent magnetic states are comprehended under the denomination of *lucidity*, or lucid vision, (Fr. *Clairvoyance;* Germ. *Hellsehen.*)

In the sixth degree, the lucid vision extends to all objects, near and at a distance, in space and time, and is hence called the degree of *universal lucidity.* No patient, it is declared, can reach the higher degrees of magnetism, without, like good masons, having previously passed through the lower. Individuals, it is true, are sometimes placed in the higher degrees at the very first magnetic treatment, but they are supposed to have previously passed through the intermediate stages in so rapid a manner, as rendered it impossible to distinguish the transitions. "In conclusion," says one of the accredited writers on the subject,* "in the higher degrees of Animal Magnetism, we may find a complete practical refutation of all the material theories of the human mind, an impressive proof of the independence of the soul, and the strongest grounds for presuming its immortality; since it has been demonstrated that in its manifestations it is not confined to any one particular portion of the corporeal organism, and that it is capable of exercising its functions without the use of any of those material organs, by means of which it usually maintains a correspondence with the external world."

This is the Magnetism of Europe. Let us see whether that of America is a match for it. To this end we shall quote from the highest authorities. Mr. Hartshorn, in the notes to his translation of Deleuze, affirms, that a female *Clairvoyante*, now in Providence, Rhode Island, can read bank bills and the superscription of letters when they are held with the blank side to her occiput! and that the fact can be substantiated by "hundreds of the most respectable citizens of his native city!" Further, a gentleman wrote a sentence, which he communicated to no one, enclosed the paper "between two thick cards, folded them up in a deep blue sheet, and sealed the whole with wafers, and wax impressed with his own signet." Notwithstanding this, the Clairvoyante read it! Another, or the same, explored the abdomen of a man a mile off, and discovered what his physician had not, that his spleen was enlarged! The man died, and on dissection in presence of eighteen doctors, the organ was found to be augmented to ten times its ordinary size! Finally, Col. Stone assures us, that one of the Providence ladies visited, in imagination, his own house in New-York,

*J. C. Colquhoun, Esq.

and gave a correct description of certain paintings and other articles in his library, some of which were purchased and placed there by himself immediately before his departure for Providence, and were not known to be there even by his own family!!! From these citations, it clearly appears, that the Animal Magnetism of the New World is quite equal to that of the Old. At the same time the means of exciting it are simplified. A Providence magnetizer seldom employs any other organ than the eye. With him, it is not an affair of manual labor. The iron rods of Mesmer and Deslon, and the *passes*, often long-continued and even fatiguing, with the hand, practised by the Marquis de Puysegur, Deleuze and Foissac, are found unnecessary, in our new and susceptible community. A stroke of the eye closes that of the magnetizee, another makes her a somnambulist, a third disembodies her spirit, and sends it on errands to the ends of the earth; we must say, however, that we have not heard of any who have yet travelled beyond India.

Having, as we suppose, sufficiently set forth the phenomena of Somnambulism and Clairvoyance, and cited some of the most reputable witnesses for their existence, let us hear what can be said on the other side, and

First. It should be recollected that the Animal Magnetism of the present century, is represented by all its advocates, as identical with that of the last, although excited by simpler means, and being more spiritual in its character; but the Magnetism of the last century was, as we have seen, overthrown and utterly exploded by the searching experimental inquiry of the French academicians. In a series of phenomena stretching out from the natural and probable, if we find the first to be fallacious, or to depend on a known and common cause, (in this case the power of the imagination, as *demonstrated* by the Parisian Commissioners,) it follows, on the soundest philosophical logic, that the more supernatural appearances are an imposture or a delusion! And on this proposition we might safely rest the cause of the incredulous; but to prevent cavilling, we shall not stop here.

Second. The great difference in the phenomena between the Magnetism of Mesmer and Deslon, in 1784, and that of the Providence Magnetisers of the present day, when these effects depend, as it is alleged, upon the same agent, shows that the magnetizees are governed by fashion, in what they display. An electric shock was the same in the time of Franklin as it is now, or will be hereafter. The relations between the living body and those subtle fluids, one of which is supposed to be the exciting cause of all the magnetic phenomena, are not variable, but constant—and the phenomena themselves should be uniform.

Third. It *might* be admitted, that the susceptibility of the eye may be so increased by natural causes, that the organ would take cognizance of lower degrees of light than in its natural state, and thus perform the function of vision under circumstances which commonly preclude it; but this is totally different in principle from a transfer of the function to some other part of the body, as the occiput, the temple, or the epigastrium, the nerves of which, (not, moreover, reached by the rays, like the retina) have their own appropriate office. The introduction into the Somnambulist, of a portion of vital power from the magnetiser ought, if it had any effect, to raise *all* the susceptibilities of the former, not derange and transpose them. It would even be less marvellous, if the *whole* surface of the body became sensible to the luminous rays. The alleged facts on which the theory is built, are not such as would occur if the theory were true.

Fourth. When the Somnambulist sees objects placed on the further side of what to all the world are opaque bodies, it can only be because the intervening objects are to *him* transparent; but transparent bodies are not in themselves *objects* of vision. They transmit the light without decomposing it: opaque bodies decompose and reflect it. But if the bodies which constitute the screen are transparent to the clairvoyante, those which are placed beyond it cannot be less so, and must, therefore, be seen through, like those which are intended to conceal them, and if seen through they cannot be recognised by the eye. Hence Clairvoyance would seem to be a physical impossibility. We of course exclude from this proposition those cases of acute natural vision, in which a dim light is sufficient for a perfect inspection, as they depend on a different and well-known principle.

Fifth. But the Somnambulist performs much higher and more difficult functions. He not only *sees* things, through opaque masses of matter, but he *comprehends* the connexions among them, their forms, the laws of their relations and dependencies, although previously unacquainted with the whole. Thus Dr. B., of Providence, employed, as we have already said, a young lady whom he had magnetized to ascertain the seat of a visceral disease, although she was ignorant of the structure of the body; and many of the European Somnambulists, of the most ignorant classes, are said to have not only done the same thing, but pointed out infallible methods of cure. Here then is not merely a transposition of the sense of sight, and an exaltation of it till opacity vanished, but the actual bestowment of a new *intellectual* power, raising the individual above the necessity of studying the organization and laws of natural bodies, and approximating her to God himself. If this were supposed or pretended to be brought about by supernatural communication, it might be received as a miracle, but when it is presented as the effect of a natural cause, most reflecting minds will, we should think, require even stronger evidence than they would require to be convinced of an influence from above.

We are not, however, under the necessity of relying on these modes of refutation. We began this article by an allusion to Mr. Durant's book on the Providence Somnambulists. Of the style and manner of this performance, we can speak in no other than terms of perfect condemnation. It is throughout coarse and even vulgar; ambitious, but vapid, in its attempts at wit; and redundant in actual abuse of many highly respectable men. Nevertheless, it bears intrinsic evidence of truth, and although many weeks have elapsed since its publication, those who are arraigned for ignorance, credulity, imposture and falsehood, have not, as far as we know, made any reply. From this we cannot excuse them on the ground of their having been treated with great indecorum. The book professes to set forth facts and experiments; and cites times, places and persons, when, where and among whom the experiments were made; and silence on the part of those implicated, either as actors or witnesses, must be received, as acquiescence in the statements given. Now, these statements amount to this, that no Somnambulist, either in New-York, where the experiments were commenced, nor in Providence, where they were ended, displayed the slightest degree of Clairvoyance, nor succeeded in guessing, beyond the ability of the most ordinary mortals. Several of them, indeed, did not succeed in maintaining their alleged insensibility to all impressions, except those made on them by the magnetiser and the persons by him placed in magnetic communication with the magnetised. One started from her chair and screamed for help, when, during the absence of her magnetizer, those around her proposed to take a quart of blood from her arm; another started when Durant suddenly clapped his hands near her head, and another, apparently forgetting herself, went into free and natural conversation with him, while he was not in magnetic communication with her, when, according to their magnetizer, she was insensible even to the sound of a cannon! He persuaded one that he had discovered a substance, that would intercept the magnetic currents, which he professed to believe flowed into her system from the eyes of her magnetizer; whereupon, regarding him as a believer in their reality, she ceased to feel their influence, and was much amused at being able to thwart the influence of the person who could generally disembody her spirit in five minutes. The screen which Durant interposed was a sheet of tissue paper! The Somnambulist on whom this experiment was made was Mrs. Andros, whose husband was her magnetiser—a seemly arrangement—and who had brought her from Providence to New-York, to exhibit her for money! Our author did not fail to attack Miss Brackett, the subject of Col. Stone's letter, but her magnetic power vanished at his approach. She performed nothing for him, whereupon he falls into a passion with the Colonel, who he informs us had been his benefactor, and abuses him to the end of the volume. As to Clairvoyance at the epigastrium, it is according to our author, effected by the eyes directed downwards along the nose, when a handkerchief is tied across the face. Whoever thus blindfolds himself will find that he can see things placed over the pit of the stomach; but it seems incredible that the scientific men of Providence should not, in their experiments, have, like the Parisian Commissioners of 1784, devised a better method of enclosing the eyes.

As a specimen of Mr. Durant's book, we shall transcribe, entire, his sixteenth experiment.

"On leaving Miss Brackett, I called, pursuant to a previous appointment, to witness an experiment with Miss Parker, at the residence of Dr. Brownell, who is her magnetizer, and a regular professor of the magnetic doctrine. Dr. Brownell is also an eminent physician, and bears an unblemished reputation for probity and honor. A short interview with that gentleman satisfied me that he was above suspicion of deception, and as sincere and honest in his belief as the other professors of Animal Magnetism.

Miss Parker* is one of the most celebrated somnambulists in this country. It was her who discovered the diseased spleen recorded in chapter four. She is about twenty-seven years of age, and in her true character, I would place her second best to all the somnambulists, Mrs. Andros being considered first for intelligence and tact in the deceptions of the craft. I had little previous conversation with Dr. Brownell about my theory. He thought he could convince me that Miss Parker possessed clairvoyance, and this experiment was expressly to satisfy me on that point. There were present Dr. Brownell, Dr. Miller, Mr. Church, Miss Parker, and myself. Brownell's process for magnetizing is the same as that of Mr. Andros. The Doctor was seated about four feet from Miss Parker, and closed her eyes in about eight minutes, when he said she was in magnetic sleep, and insensible to any torture or noise, except from one in magnetic communication, and perfectly subject to his "will" in all her actions. She is never blindfolded, because she does not complain of the light. Her invisible eyes are generally on top of her head, though sometimes she sees best behind. She always keeps her eyelids a little open, in order to catch a glimpse of the object before it goes on top of the head or behind her. This is very important, because sometimes the name of the object is not spoken aloud, and then it would be impossible to acquire the necessary knowledge by the magnetic cords through the ears. She is very shrewd, but she found my theory too deep for her shrewdness, as you shall see by the particulars of this experiment. She was seated in a rocking chair, directly in front of the sofa, on which sat Miller, Church and myself. I requested the doctor to "will" the hand to raise, but he could only move it an inch or two. He remarked, this was extraordinary, because he could generally "will" the hand to touch the head.† I concluded to try the same game which I tried with Miss Brackett, and asked Brownell if she could hear what I said. He replied, "no, she can hear nothing unless I will her to hear." I held a knife in my hand, and said audibly, "see if she knows I have a knife in my hand, "What has Mr. Durant got in his hand?" "*A knife.*" "See if she can tell that I have a book in my hand." "What has Mr. Durant got in his hand?" "*He holds a book.*" "Yes, now we will try the other part of my theory, doctor; and in this case you should not know yourself what I hold. Ask her what I have in my hand." I held a piece

---

*The first of this lady's fame was having four artificial teeth inserted while in magnetic sleep, without being conscious of it until she was waked. This case is often used in support of the doctrine; the true theory is this: she wanted four teeth, and suffered excruciating pain without complaining, for the sake of having the teeth inserted gratis. She is poor, and I fear will remain so, unless she tries some new science that my theory cannot unravel.

†The true cause of failing to get the hand up, was, that both she and Miss Brackett had heard of my theory working miracles on Miss Ebon, and they concluded to thwart me in that, at least. Miss B. and Miss P. are great cronies; they come together almost every day, and tell each other what has transpired. By that means they obtain much knowledge in the way of "clairvoyance;" these two have been magnetized together, and very interesting stories are told of their "*monkey shines*" in such cases.

of pine chip behind her. "What does Mr. Durant hold in his hand?" She hesitated some time, and said, "*It is paper.*" "Ask her what colored paper." "What is the color of the paper?" "It is blue, nearly white." "Are you sure it is paper?" "*Yes.*" "Is there any writing on it?" "*There is some letters on it, but I cannot see what they are.*" These and similar questions were repeated at least twenty times, and she continued to give similar answers. I held it perfectly enclosed in my hand to her head, face, and various other parts; but still, "it was paper—light blue—nearly white—some letters on it." "What is the use of it?" "*I can't tell.*" "What is the shape of it?" "*Long, and appears to be in two parts.*" I now held it up open over her head, so that Miller, Church and Brownell could see it. Still "*it is paper—nearly white—light blue—some letters on it—can't tell what they are—it is in two parts.*" "Which hand is it in?" "*Right.*" It was in the left. I now shifted it to the right hand. "Ask her if she is sure it is in the right hand?" "Are you certain it is in the right hand?" "*It was then in the right hand, but it is now in the left.*" So she continued for more than a half hour, asserting the pine chip was "paper—light blue—writing on it—in two parts," &c. I clapped my hands suddenly, and she started as much as a person awake would do. "How is that, Doctor; she hears no one but you, and yet she starts at the noise I make?" "Oh, she can't be sound asleep." "How do you know when she is sound asleep?" "We know they are sound asleep when they tell correctly, and do not start at a noise, was his reply. "Well then, Doctor, she cannot be sound asleep, for she tells all wrong. You had better magnetize her more;" so the poor doctor went to work magnetizing her more, and asked her if she felt perfectly asleep. "*Yes, I am perfectly asleep now,*" was her reply. I clapped my hands, and she started again; "how is that, doctor, she cannot be asleep yet?" "Well, I don't know, she never started so before;" and here the doctor took up two books and clapped them together; she moved very little, and he tried it again, when she scarcely stirred* a muscle; "I think she is perfectly asleep now," said the doctor; "take the books and see if you can start her;" I declined, and expressed myself satisfied that she was now asleep. Dr. Miller remarked, "suppose you try if she can read; she does read in her sleep, don't she, doctor?" "Yes, she can read, but she can always tell one letter best; suppose you step in the other room, Dr. Miller, and put a large letter in a book, and hand it to me. I will see if she can tell what it is." Dr. Miller accordingly arranged a large letter in a book, and gave it to the doctor, who remarked, "the letter must be upright." so Dr. Miller opened the book to be sure, and gave it to him, and he asked her to look at the letter, and tell what it was; after a little hesitation she answered, "*it is M; I think it is M; but I can't see very plain.*" "Ask her to be sure." "Are you sure it is M?" "*Yes, it is M.*" He opened the book, and what do you suppose it was? a large D, at least one inch long. I pitied the poor doctor, and feared he would be obliged to believe my theory; he could not account for so

*She was, by that time, prepared to stand any noise.

many failures. I asked the doctor if she could distinguish flowers; and proposed trying her, which he consented to; and while I was taking some flowers from a boquet, in the adjoining room, to cover a bottle of hartshorn, he came in; I remarked, "we will see if I can cheat her." He made no objections; so I put it to her nose. She immediately opened her mouth, and ceased breathing at the nose; but she stood it well for nearly half a minute; when the doctor thought it might strangle her, and wishing to keep him in good humor, I took it away. At that moment Dr. Miller remarked, "there are tears in her eyes; she is crying about something." "*Oh, see how the man is whipping the child,*" was her shrewd excuse. "She sees a man whipping a child,"* said the doctor; who continued, "she often laughs and cries at things which she sees in her sleep." Dr. Miller proposed asking her the time by the clock. Dr. Brownell said, "try your watch, Mr. Durant, alter the hands, and I think she will tell the time by it; she seldom fails in that." I did so, and held the face to the palm of my hand. Dr. Brownell asked, "what time is it by Mr. Durant's watch?" She hesitated a little, and opened her mouth with a low whisper. "What do you say? speak louder; we don't hear you." She still continued to move the lips, as if replying to each question. Once the doctor said, "I think she says something about ten and three."† "Well, be sure you know what she does say; my ears are very sensitive, and although close to her lips, I cannot distinguish a word." "Speak louder." "I don't hear a word you say." This pantomimic farce continued more than half an hour, when he gave up, and said, "I don't hear what she says; it is no use to question her any more; she never acted so before, and I don't know

*Recollect, she had not been out of the room, even in spirit; her last ramble was, guessing *at* a letter in the room. They are all very shrewd in this particular generally; if they are away describing furniture, and you suddenly ask them to look at something in the room, they will say, you must bring me back first; I can't come back alone. But she forgot herself in this particular instance; her excuse to be consistent with the doctrine, should have been, "*Doctor, your room is full of strange smoke; I can't see it, and yet it strangles me,*" or any excuse relating to the room which she was in.

†Here is a proof of her shrewdness, and a key to Col. Stone's pictures. She knew by my conversation with the doctor, that she had failed in every thing, and she knew the chance of guessing the time was not more than one in one thousand, and though she would not care much about this failure alone, yet this connected with the others, she knew would be a strong proof of her imposition, and therefore, she articulates something to sound like *three o'clock*, and ten minutes; but so low, that it might be taken for any thing else. Now, suppose the time by the watch was thirteen minutes past one, and all present, including myself, were believers in the doctrine. I would then have said, "ask her again, and see if the ten is not *one*," as that sounds like ten. She would hear it, and when asked, "speak louder; I can't hear you," she would have said, "* * * *one* * * *" "yes, I think she says one;" "well, that is correct; it is thirteen minutes past one;" "but she did not tell the minutes correct; see if you was correct about the three;" "speak louder, we can't hear you; what time is it?" and she would have answered, "*thirteen* * * * *" "yes, she says thirteen," and all present would have gone to a justice of the peace to make affidavit, that Miss Parker told the time by a watch, with the hands altered, when it was enclosed in paper, or at her back; but my theory was too deep for one who can see a *diseased spleen.* The watch was twenty minutes past four, and she would not have told it in four times twenty guessings.

the cause, unless it is something in the atmosphere about you."* "Well, Dr. you will acknowledge she has failed in every thing with me, except the book and knife, which you knew† yourself; so you must give up all pretensions to seeing with invisible eyes." The doctor acknowledged again having failed with me; but he cited the case of diseased spleen, Col. Stone's letter, and talked about respectable proof of what had been done. I told the doctor I was now through with all the principal somnambulists in Providence, and that they had all failed as Miss Parker had failed; but that I would stay a week or a month longer, if he would name a time to show me another experiment. He expressed sorrow both in words and looks, that every somnambulist had failed with me, but could not give another experiment, because Miss Parker had already spent too much of her time in these experiments; he felt reluctant to ask her for another. She had been waked up, and was then in an adjoining room, where Miss Brackett had just arrived on an afternoon call. I heard a part of their conversation. It was about an article on Col. Stone's letter which she (Miss B.) had just received in the N. Y. Commercial, of the 4th Sept.; they had a fine time over it; but I could not avoid a thought of the future, and, in imagination, I saw Miss Parker weeping over this narration, (for she has some virtues and some modesty;) and I imagined myself saying to her, Weep, woman, weep, for your dark deeds are unveiled; weep for the disgrace you have brought on yourself; weep for the stain which your deception has caused to your sex; weep tears of repentance to wash out your iniquities, registered in heaven; weep, woman, weep tears of humanity, till your heart melts within you, for "*Oh, see,*" I am now lashing "*the child!*."

With this quotation we leave M. Durant, having made it with reluctance, and from the necessity of the case.

Let us now turn our attention to the Somnambulism and Clairvoyance of Europe. While composing this review, we received, in our London Medical Gazette, a new Report to the Royal Academy of Medicine, as late as the 22d of August last, by a committee of its own body. This committee, appointed six months before, consisted of Roux, Bouillaud, Cloquet, Emery, Pelletier, Caventou, Cornac, Oudet, and Dubois, several of whom are known to our readers as men of eminent talents and philosophical acumen. The immediate cause of the inquiry with which they were charged, was a letter addressed to the Academy by Dr. Berna, a Parisian physician, professing to demonstrate to them the reality of Magnetic Somnambulism and Clairvoyance.

In his *programme* he engaged to prove the following points:

*Several of the professors attributed the total failure of all experiments during the week I was at Providence, to a "*bad atmosphere about Durant.*"

†I should think it strange, if she did not know those two things, when she had her *ears* open, for I spoke the names loud enough to be heard by an adder.

"1st. Somnambulism.

2d. Proof (constatation) of insensibility to pricking and tickling.

3d. Restitution, by the mental will, of the sensibility.

4th. Obedience to the mental order to lose motion.

5th. Obedience to the mental order, to cease answering in the midst of a conversation; and to the mental order, to answer again.

6th. Repetition of the same experiment, the magnetizer being separated from the somnambulist by a door.

7th. Waking.

8th. According to the mental order which shall be enjoined in the somnambulic state, persistence in the restoration of the sensibility, and also persistence of the power of losing or recovering this sensibility at the will of the magnetizer."

For this purpose he selected two patients, or rather subjects, which we may suppose he regarded as his most favorable specimens. The first was a young girl; the second a woman, thirty years of age. We shall not follow the committee through the details of their experiments, which, like those of Dr. Franklin and his associates, in 1784, were conducted with great caution and fairness, but content ourselves with transcribing a part of their conclusions.

"*Resume and Conclusions.*—1st. It results, in the first place, from all the facts and all the incidents of which we have been witnesses, that in the preamble no special proof was given us of the existence of a particular state called that of magnetic somnambulism; that it was only by assertion, and not by demonstration, that the magnetizer proceeded in this matter, affirming to us at each sitting, and before any experimental trial, that his subjects were in a state of somnambulism." * * *

2d. According to the terms of the programme, the second experiment was to consist in the proof of the insensibility of the subjects. But after having mentioned the restrictions imposed on your Commission—that the face was put out of sight, and removed from every trial of this kind—that it was the same for all the parts naturally covered, so that there remained only the hands and neck;—after having reminded you that on these parts we were permitted to exercise neither pinching nor scratching, nor the contact of any body, either on fire or of a slightly raised temperature, but were limited to the sticking in of needles to half a line deep; after recalling all these restrictions, we are justified in deducing from these facts—1st, that none but very slightly painful sensations could be excited; 2d, that these could be produced only on parts perhaps habituated to this kind of impression; 3d, that this kind of impression was always the same, and that it resulted from a kind of *tatonage;* 4th, that the features, and especially the eyes, where painful impressions are more especially indicated, were hidden; 5th, that in consequence of these circumstances, an *impassibilite*, even complete and absolute, could not be a conclusive proof to us of the abolition of sensation in the subject in question.

3d. The magnetizer was to prove, that by the mere intervention of his will, he had the power of restoring, either totally or partially, sensibility to the somnambule. * * * * All the trials made for this purpose, completely failed. * * * *

4th. What we have said of the abolition and restitution of sensibility may be completely applied to the pretended abolition and restitution of motion: not the slightest proof was given of it to us.

5th. One of the paragraphs of the programme had for its title—Obedience to the mental order, to cease in the middle of a conversation from answering verbally, or by signs, to a particular person.

The magnetizer endeavored, in the sitting of the 13th of March, to prove that the tacit power of his will could produce this effect; but it results from the facts then observed, that, far from producing this result, his somnambule appeared no longer to understand, when he did not wish to prevent her from understanding; and that she seemed to understand anew, when positively he did not wish her to understand. * *

6th. Transposition of the sight. Yielding to the wishes of the Commission, the magnetizer, as you have seen, left the abolitions and restitutions of sense and motion to pass to greater facts—that is, to facts of vision without the assistance of eyes. * * *

As to real facts to prove the vision by the occiput—absolute, decisive and peremptory facts—not only were they wanting, and completely wanting, but they were of a nature to give rise to suspicions as to the honesty of this woman. * * * * *

7th. *Clairvoyance.*—Despairing of proving the transposition of the sense of sight—the nullity and superfluity of the eyes in the magnetic state, the magnetizer wished at least to take refuge in the fact of *Clairvoyance*, or vision through opaque bodies.

You know the experiments made on this subject; they present the capital conclusion, that a man placed before a woman cannot give her the power of seeing through a bandage. * * *

If, now, you ask what ultimate and general conclusion we would draw from the whole of the experiments made under our inspection, we would say, that M. Berna was without doubt under an illusion, when, on the 12th of February, in this year, he wrote to the Academy of Medicine, stating, &c. Those facts which he promised to show are all known to you; you know, as we do, that they are any thing but conclusive in favor of the doctrine of Animal Magnetism, and that they can have no relation either with physiology or with therapeutics."

The whole committee, it will be observed, subscribed these conclusions, while one of them, Oudet, is the very person, as we learn from the Report, who had previously testified in the Academy, that he had extracted a molar tooth from a female in a state of Somnambulism, without her being conscious of it. The committee, then, was not composed entirely of the opponents of Animal Magnetism.

To this testimony we shall only add, from the London Medical Gazette for the 16th of September last, that a Magnetizer, in the presence of a large number of medical gentlemen, had just befo undertook to magnetize several patients in the Middlesex Hospital, and repeated his operations for several days. He selected some "hysterical girls on whom to try his powers, but entirely in vain—they were scarcely even frightened."

Here we shall leave the subject of testimony. To establish that which is at variance with the experience of the world, it is necessary that all the witnesses should concur. In regard to Somnambulism and Clairvoyance, the evidence is not only divided, but that of the most scientific cautions and disinterested, is against it, and to our minds carries irresistable conviction.

But is there *nothing* then in Animal Magnetism? Are all who now stand forth as its advocates, and their predecessors, either impostors or the dupes of impostors? Is such a theory of the case allowable? We think not. We do not believe that they who first promulgated the doctrine, invented the phenomena; and before we can have a counterfeit, there must be a reality. Imposture, it is true, need not limit itself to counterfeiting, but may go far beyond its prototype.

We suppose, then, that one human being may and often does exert over another a very great influence, by looks and movements, merely addressed to the eye. The power of snakes and cats over birds, is a generally acknowledged fact in natural history, or at least the testimony in its favor is highly respectable; and even the ferocity of a dog may be subdued and converted into cowardice by the eye of man. When Dr. Willis was censured in the British House of Commons, for allowing his patient, George III, partially insane, the use of razors to shave himself, his defence was, that he could have prevented the king from committing suicide; and on being asked by Mr. Burke, how? that experienced physician replied, with my eye—at the same time directing it upon the celebrated orator, with such an expression, as compelled him to turn his head aside, and silenced further interrogation. It is known to all the world, that a glance of the eye, on a first interview, has fallen upon a young man, with such fascinating power, that he has never afterwards been able to extricate himself from the influence of her who sent it forth. The intensity of the intellectual character is strikingly manifested in the stroke of the eye, and it is an axiom in practical magnetism, that the operator must have an intellect superior to that of the patient. Here then we take it, is the starting point and a reality for many of the phenomena to rest upon. But does any thing physical pass from the eye of the magnetizer to that of the magnetizee? We believe not. The agent is an expression of the mind in the language of the eye, the effect is produced on the mind of the patient, and thence transferred to the body. Emotions are raised, and the imagination is excited, and immediately exalts the emotions still higher. The nervous system becomes agitated, and re-acts upon the mind, and thus a certain number and variety of phenomena are produced. The nature and true cause of these phenomena may not be understood by the parties concerned, and then the theory of a mysterious physical influence comes into their aid.

In the second place, certain manipulations are performed. Gesture, not less than occular expression, is a universal language. There is therefore a susceptibility in the human mind to be acted upon by it. Gesture can raise in us natural emotions. If the gesticulation be strange and unnatural, and the eye should at the same time give evidence of intensity and earnestness of purpose or desire, a new and incomprehensible emotion will be raised in the person towards whom it is directed. His curiosity is excited, a feeling of alarm begins to germinate in his mind, he becomes perplexed, and at length mystified.

Third. Credulity in regard to the existence of occult influences, both natural and supernatural, is a trait of human nature. It exists in very different degrees in different minds, and although most conspicuous in the ignorant and weak-minded, is by no means, universally, in an inverse proportion to the intellect. It existed in great activity in Dr. Johnson, who believed that he had once heard his mother, when he was at Oxford and she was in Litchfield, call him *Sam!*

Fourth. Now as life is a mystery even to the profoundest physiologist, they who are ignorant of its laws are predisposed to believe in all that may be affirmed, by those whom they presume have studied it. Let such an one then be subjected to the steady and unearthly gaze of a magnetizer, and watch his new and strange gestures, and, it is quite impossible that an agitation of mind and body should not be excited, and this once experienced, must confirm the wavering belief of the patient, and greatly augment the influence of the subsequent experiment.

Fifth. The nature of this effect, or the particular mode in which it is manifested, will be modified by circumstances. When a solitary individual is subjected to the process employed at the present time, the silence of the room, his fixed position, his required impassiveness, the steadiness of the eye of the magnetizer, and the monotonous movements of his hands and fingers, are calculated to produce drowsiness; and this effect once produced, a new principle reinforces those gentle somniferous influences. Imitation or fashion is not confined to an apeing of that which passes before us, though it is then most active, but exerts an influence at a distance. The effects which the patient understands have been produced in others, he encourages or invites in himself, and, it is they, which are most likely to occur. Hence drowsiness or sleep will naturally be one of those nervous affections, which may be legitimately produced by the means which we have been considering. In the days of Mesmer, convulsive movements were more common than sleep; for he assembled and connected his patients together, by three different ties. They kept each other awake, as mere association will, and the iron rods which he employed for conducting a physical agent from the earth into their limbs, suggested the idea of convulsive action; finally, when this occurred in one of the circle, imitation, as the Commissioners observed, seemed to assist in propagating it successively and rapidly to all the rest.

Sixth. We believe then that the nervous function generally, and the feelings and intellect of a certain class of patients, may be excited and disturbed by the processes which the magnetizers employ, without the transmission from them of any thing whatever; and that these effects, which all parties honestly believe to be the offspring of some occult cause, are the prototype of the whole display which is made in the publications by the magnetizers. It is these which the magnetizers counterfeit or exaggerate. To what extent this imposture is carried, is a moral not a physical inquiry; but not to enter upon it, we do not hesitate to express the opinion, that immense impostures have been practised. Both the opportunity and inducement exist, and it would argue great incredulity to believe that they do not invite to it. The extent to which the actors may be parties to these impositions, is various. The magnetiser may be honest, and the magnetisee dishonest—they may be transposed in this respect—or they may act in collusion.

Seventh. Animal Magnetism, then, is resolvable into the excitation of the feelings and the imagination, by the means which are prescribed and employed by the magnetisers, and which, as we have seen, vary considerably in different ages and nations. To what extent this excitement may be raised, and in what degree it may be made to influence the functions of the nervous system, can only be determined by observation and experiment; but, that very striking effects may be produced by it, cannot be doubted. In weak and badly balanced minds, it might occasion mental alienation, for all strong feeling, in connexion with excited imagination, is apt to disorder such minds. And, indeed, we are disposed to think, that much of what has been related, by some of those who have been thus excited, was, indeed, from true but temporary insanity. Their imagination imposed upon them a delusion. They announced their vivid maniacal conceptions for realities, and believed in the truth of what they stated. In feeble and nervous constitutions, the physical effects of this mental action might be spasms of the muscles, disturbed, exalted or benumbed sensibility, reverie, drowsiness, and for aught we know or believe, more or less of Somnambulism—but neither the capacity of seeing through opaque bodies, nor half round the globe; though it *might be* a conviction on the part of the *patient*, that he could do both. In certain forms of nervous disease, this excitement of mind may produce beneficial effects; but the extent and kind of influence cannot be foretold; and while we recognize artificially excited states of feeling as among the legitimate resources of the profession in the treatment of these maladies, we must be excused from believing, that Animal Magnetism will ever become an available portion of our materia medica. Nevertheless, a single cure, now and then performed, will keep alive the popular delusion, and even draw in a portion of the medical profession.

Eighth. How does it happen that Animal Mognetism exerts its influence epidemically? We have seen a hiccough in a nervous girl. excite to an unnatural degree the same affection in her two female companions. Chorea is well known to become epidemic in boarding schools. Yawning will spread from a single individual throughout a whole circle. Particular modes of medical practice sometimes spread through a whole community of physicians. Speculation in stocks or property frequently pervades a city. Finally, a particular superstition, as that of witchcraft, has often taken as entire possession of great masses of people, as if it had been an epidemic disease. In all of these cases, every new believer creates others—"what every body believes must be true"—all become involved in the mists of credulity—the aggregated or social mind falls into disease, and if no extraneous influence is brought to bear upon it, there is no remedy but to wait, as when medicine is withheld in the diseases of the body, till the morbid action wears itself out and subsides. Sympathy, imitation, love of the marvellous, the very confidence of man in man, without which society could not exist, the desire of interchanging opinions and feelings, and their reciprocal influence on individuals, are natural causes sufficient to explain the spread of Animal Magnetism through a community, as it is now raging in Providence, as it has at different periods prevailed in the cities of France and Germany, and may, perhaps, at this time, be spreading in London.

Ninth. We know that caloric passes from one animal to another, by contact and conduction. We also know that the human body is a good conductor of electricity, and that it may be made to modify or to assist in the performance of some of our functions, both animal and organic; still further, we know that a change in the temperature of our bodies, or any part of them, affects the state of the electricity which is in or around them; effects which belong to the head of thermo-electricity, in the science of natural philosophy; finally, we know that the true magnetic phenomena, which are displayed in the magnetic world, are curiously modified by electricity; but nothing yet known of the effects of any of these agents upon the living system, warrants the conclusion, that the phenomena of Animal Magnetism are produced by any one or all of them. On the contrary, the highest degrees of these phenomena are said to be produced under conditions which demonstrate, that these active agents are not pressed into the service of the magnetizer, as for example, when he only looks at the person whom he magnetises, or exerts his influence without being present. D.

